# Experimental and numerical investigations on spray characteristics of fatty acid methyl esters

**DOI:** 10.1098/rsos.171121

**Published:** 2018-02-21

**Authors:** R. D. Lanjekar, D. Deshmukh

**Affiliations:** Spray and Combustion Laboratory, Discipline of Mechanical Engineering, Indian Institute of Technology Indore, Indore, India

**Keywords:** biodiesel, spray tip penetration, fatty acid methyl esters, OpenFOAM

## Abstract

A comparative experimental and numerical study is conducted to establish the significance of the use of single-component over multi-component representatives of biodiesel, diesel and their blend for predicting spray tip penetration. Methyl oleate and methyl laurate are used as single-component representative fuels for biodiesel. The pure components *n*-heptane, *n*-dodecane and *n*-tetradecane are used as single-component representative fuels for diesel. Methyl laurate is found to represent biodiesel of coconut, whereas methyl oleate is found to represent biodiesel having high percentage of long-chain fatty acid esters. The spray tip penetration of methyl oleate is found to be in good agreement with the measured spray tip penetration of karanja biodiesel. The spray tip penetration prediction of *n*-heptane fuel is closely following diesel spray tip penetration along with that of *n*-tetradecane and *n*-dodecane. The study suggests that the knowledge of the single-component representatives of biodiesel, diesel and their blend is sufficient to predict the spray tip penetration of the corresponding biodiesel, diesel and their blend under non-evaporating environment.

## 1. Introduction

The rise in the use of fossil fuel in the transportation sector has an adverse effect on the environment and human health. There is a need to search for an alternative renewable fuel to reduce consumption of petroleum-based fuels and engine emissions. Biodiesel is one of the alternative renewable fuels for compression ignition (CI) engines [1–3]. The performance of a CI engine fuelled with biodiesel is observed to be similar to that of the engine fuelled with diesel. However, it has a major disadvantage of higher NO_*x*_ emission than conventional diesel fuel [4–7].

Researchers have proposed several strategies to control NO_*x*_ emission from biodiesel fuel including exhaust gas recirculation, multiple injections and optimization of injection timing. Various theories are given for observed higher NO_*x*_ emission of biodiesel, such as higher adiabatic flame temperature due to fuel bound oxygen, advanced injection timing and combustion phasing. These theories for the NO_*x*_ emission are closely related to physico-chemical properties of biodiesel which are the aggregate behaviour of their fatty acid ester composition [8–10]. Several researchers have investigated the spray characteristics of biodiesel in addition to performance, combustion and emission studies. The spray characteristics study is found to be important, as these characteristics govern the air–fuel mixture formation, ignition, combustion and nature of the pollutants formed. The most commonly studied spray parameters such as spray tip penetration, cone angle and mean droplet diameter are also found to be related to the physical properties of biodiesel, which are further a function of their fatty acid ester composition [7,9,11,12]. Biodiesel is composed of various fatty acid methyl esters (FAMEs) with carbon chain length varying from 12 to 22 [7,13,14]. The physical and chemical properties of individual FAMEs govern the biodiesel spray characteristics. Allen *et al.* [15] measured Sauter mean diameter (SMD) of five different biodiesels under atmospheric conditions. Furthermore, the viscosity, surface tension and atomization characteristics of 15 commonly used biodiesels were predicted and the atomization characteristics found to be similar. The similarity in atomization characteristics of commonly used biodiesel was attributed to the long-chain unsaturated fatty acid esters. The long-chain fatty acid esters such as methyl oleate may be responsible for the higher viscosity and hence larger SMD for the commonly used biodiesel than that of the diesel [15]. The medium-chain length components such as methyl laurate may be responsible for the lower viscosity and hence smaller SMD for the coconut biodiesel compared with that of diesel fuel. On the similar lines, Ejim *et al.* [16] with statistical analysis show that palm, soya bean, cottonseed, peanut and canola biodiesels exhibit similar atomization characteristics with no significant difference in their SMD. The comparison of non-evaporating spray characteristics of biodiesel, diesel and their blends has been carried out by various researchers [17–19]. Gao *et al.* [20] have observed similar spray tip penetration for the three biodiesels jatropha, palm and waste cooking oil, which is attributed to their similar viscosity and density. Mancaruso *et al.* [17] reported similar spray characteristics for biodiesel such as rapeseed and soya bean. From the various studies, the observed similar atomization characteristics for commonly used biodiesel may be attributed to the similar fatty acid ester composition of biodiesel. Jiaqiang *et al.* [21] numerically studied biodiesel combustion and emission characteristics in accordance with their fatty acid ester composition. The biodiesels, such as rapeseed, sunflower, soya bean and cottonseed biodiesel selected for the study, differ in their degree of saturation. The degree of saturation and kinematic viscosity are observed to be related to ignition delay (chemical and physical) which consequently determines the combustion and emission characteristics. Balaji *et al.* [22] investigated spray characteristics of waste cooking oil biodiesel and its 20% blend with diesel fuel. The blend spray characteristics of biodiesel were found to be similar to those of diesel fuel; however pure biodiesel exhibits inferior poor air–fuel mixing which was attributed to the poor atomization. The experimental investigations for establishing the role of pure fatty acid esters for observed spray characteristics of biodiesel under non-evaporating environment are limited.

The present investigation is an attempt to show that the observed spray tip penetration of biodiesel may be due to their pure fatty acid ester composition. Methyl oleate is used as a single-component representative for commonly used biodiesel such as karanja, whereas methyl laurate is used for coconut biodiesel. The pure components *n*-heptane, *n*-dodecane and *n*-tetradecane are used as single-component representatives for diesel. Furthermore, the spray tip penetration prediction of single pure component and multi-component representatives is compared with biodiesel, diesel and their blend.

## 2. Experimental set-up and numerical methods

**Figure 1.**
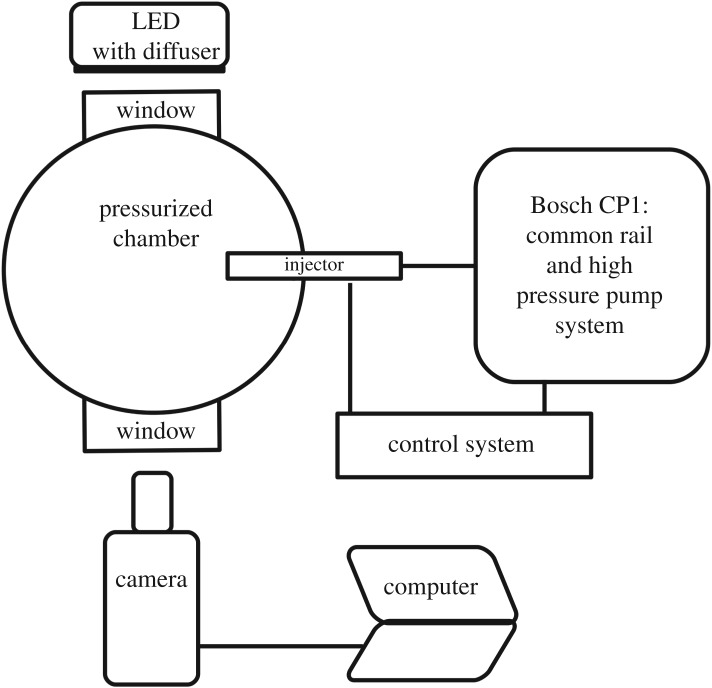
Schematic of the experimental set-up for spray imaging using backlight illumination.

**Figure 2.**
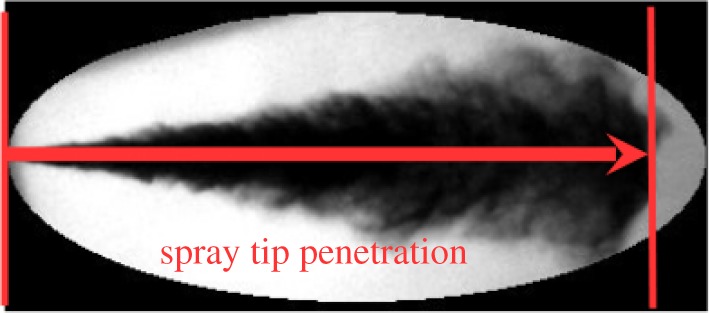
Definition of spray tip penetration.

The experimental set-up consists of a high-pressure chamber with an optical access, a high-pressure fuel injection test rig and a high-speed shadowgraphy arrangement. The schematic of the experimental set-up is shown in figure 1. The high-pressure chamber has an internal diameter of 204 mm and height of 260 mm. An optical access to the chamber for visualization is provided through rectangular quartz windows of the size 70 mm×50 mm. A light-emitting diode (LED) light with a diffuser plate is used to get a uniform backlight illumination for the shadowgraphy. A high-pressure spray injection test rig is built using common rail direct injection (CRDI) system components to inject pressurized fuel at a desired fuel injection pressure and injection duration. A seven hole solenoid injector with a nozzle diameter of 130 μm is used for the study. Six holes of the injector were blocked with microwelding to study the spray from a single nozzle hole. The fuel injection pressure can be varied from 200 bar to 1500 bar with the present set-up. A National Instruments fuel injector driver module is used to operate the solenoid fuel injector. The fuel metering of the CRDI system is governed using an in-house developed Arduino controller to maintain the desired injection pressure. A high-speed camera is used for imaging the fuel spray evolution with a sensor region of interest of 444×182 pixels and a resolution of 0.1 mm pixel^−1^. The images are acquired at a rate of 30 000 frames s^−1^ and an exposure time of 1 μs. The optical spray chamber is filled with N_2_ gas at an ambient temperature to ensure an inert environment for the spray. The spray experiments are performed for pure components, such as *n*-heptane, methyl oleate and methyl laurate. These fuels are selected as follows: the *n*-heptane represents diesel, methyl oleate represents biodiesels of karanja, rapeseed and soya bean and methyl laurate represents biodiesels of coconut and palm kernel. The pure components used in the study are purchased from TCI Chemicals with purity of laboratory grade. The important physical properties of fuels are compared in table 1. The density, viscosity and surface tension of karanja biodiesel are similar to those of methyl oleate, while properties of coconut biodiesel are similar to those of methyl laurate. The *n*-heptane, *n*-dodecane and *n*-tetradecane are also studied as these are extensively used as representative of diesel in experiments and simulations. The spray tip penetration of pure components is measured at fuel injection pressures of *P*_inj_=1000 and 1500 bar and at ambient gas pressures of *P*_amb_=30 and 40 bar. The spray tip penetration, the farthest distance between the spray tip and the nozzle, is shown in figure 2. The raw images are processed with an in-house developed image processing algorithm to get the spray tip penetration. The variation in the spray tip penetration of 6 spray events is within 3% of mean spray tip penetration.

**Table 1.** Fuel properties of biodiesel, diesel and their pure components [23,24].

	fuel properties		
	density at 288 K	kinematic viscosity at	surface tension at
fuels	(kg m^−3^)	313 K (mm^2^ s^−1^)	313 K (mN m^−1^)
methyl oleate	877	4.57	30
methyl laurate	872	2.43	28
*n*-heptane	690	0.5	18
*n*-dodecane	795	1.9	28
*n*-tetradecane	771	3.0	27
diesel	825	2.6	23
coconut biodiesel	876	3.14	22
karanja biodiesel	883	5.04	29

The numerical study is performed with the open-source computational fluid dynamics (CFD) code OpenFOAM. The geometry for the spray study is a constant volume rectangular cuboid with a base of 20 mm×20 mm and a height of 100 mm. The mesh size of the geometry is 1 mm along the height of the vessel and 0.5 mm along the width. The fuel injector with an orifice diameter of 130 μm is positioned 0.5 mm below the top of the cuboid directed downwards along the central axis. The combustion and chemistry modules of the code are deactivated to study the spray under non-reacting and non-evaporating condition. The pure components, methyl oleate and methyl laurate are implemented in the ‘OpenFOAM’ fuel library. The thermophysical properties required for the fuel implementation are obtained from the National Standard Reference Data System (NSRDS), Design Institute for Physical Properties (DIPPR) project 801 [23].

The experimental spray tip penetration is used to calibrate the spray simulation models of OpenFOAM CFD code. These calibrated models are then validated under different operating conditions. The model is further used to study spray tip penetration of various biodiesels with the single-component and multi-component representatives.

## 3. Results and discussion

**Figure 3.**
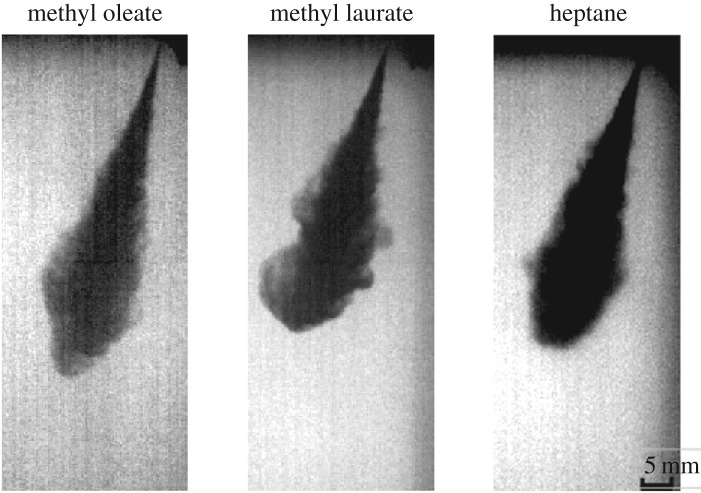
Spray structure of different fuels at an injection pressure of 1500 bar and a gas pressure of 40 bar at 400 μs from the start of injection.

The high-speed shadowgraphy images of pure component FAME and *n*-heptane fuel are acquired at different injection pressures and ambient gas pressures. The spray structure of the methyl oleate is observed to be compact and penetrating longer compared with that of the *n*-heptane and methyl laurate. The instantaneous images of these sprays at around 400 μs after the start of injection are compared in figure 3. The spray structure for methyl laurate and *n*-heptane is similar including cone angle and spray tip penetration. The spray cone angle for methyl oleate is narrow, compact and spray tip penetration is higher. The spray structure indicates more dispersion in sprays of methyl laurate and *n*-heptane.

### 3.1. Spray tip penetration

It is difficult to have one common single-component representative for all the biodiesels as their composition differs depending on their origin [25,26]. Hence, it is important to know whether the selected single-component representative has similar spray characteristics to that of the biodiesel. The spray characteristics of individual pure component help to understand their effect in multi-component biodiesel spray behaviour and also to identify the dominant component. In the present study, methyl oleate is selected to represent soya bean, karanja and rapeseed biodiesel and methyl laurate for coconut and palm kernel biodiesel [24,27].

**Figure 4.**
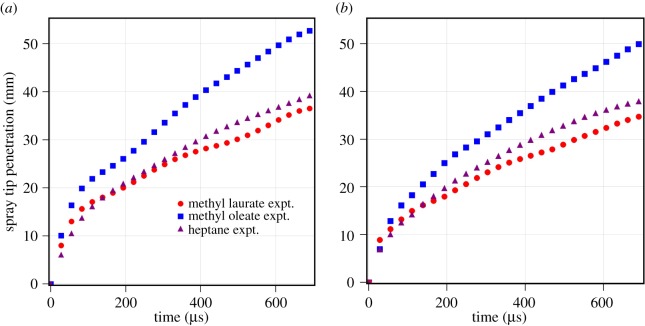
Measured spray tip penetration of methyl laurate, methyl oleate and *n*-heptane. (*a*) *P*_inj_=1500 bar and *P*_amb_=30 bar. (*b*) *P*_inj_= 1500 bar and *P*_amb_=40 bar.

The biodiesel has a range of the fatty acid esters, from lauric to erucic with a number of carbon atoms ranging from 12 to 22, present in different proportion. The effect of chain length on spray tip penetration is shown in figure 4. The spray tip penetration is compared at fuel injection pressure of *P*_inj_=1500 bar and at ambient gas pressures of *P*_amb_=30 and 40 bar. The spray tip penetration of methyl oleate is higher than those of methyl laurate and *n*-heptane at both the ambient gas pressures. The spray tip penetration of *n*-heptane and methyl laurate is similar at both the gas pressures. This indicates that the atomization behaviour of methyl laurate and *n*-heptane is identical. Moreover, there is a small increase in spray tip penetration of *n*-heptane and methyl laurate with a decrease in ambient gas pressure from 40 bar to 30 bar. The spray tip penetration for methyl oleate increases by more than 20% with a decrease in ambient gas pressure from 40 bar to 30 bar. This may be due to the poor atomization of methyl oleate which may lead to larger droplets at lower gas pressures leading to longer spray tip penetration.

The spray tip penetration in figure 4 also represents the effect of chain length of FAMEs. Methyl laurate has 12 carbon atoms and methyl oleate has 18 carbon atoms in their molecules. The long fatty acid chain length methyl oleate shows a longer spray tip penetration than methyl laurate irrespective of the operating conditions. The physical properties of methyl oleate, such as kinematic viscosity, surface tension and density, exhibit higher values than those of methyl laurate. The kinematic viscosity of methyl oleate is 4.57 mm^2^ s^−1^, which is around 50% higher than that of methyl laurate. Similarly, the surface tension of methyl oleate is 6% higher than that of methyl laurate. The spray tip penetration of methyl oleate is observed to be 15 to 30% longer than that of methyl laurate. These physical properties, specifically kinematic viscosity and surface tension, govern the mean diameter of the spray through atomization [15]. The higher viscosity and surface tension of methyl oleate tend to produce spray with bigger droplets and hence higher momentum. The spray with higher momentum droplets is able to penetrate longer, as seen for the case of methyl oleate. The density of the fuel affects the mass flow rate profile and hence the injected mass per unit time. Hence, the fuel with higher density injects more mass per unit time, which will be valid for methyl oleate having higher density than methyl laurate. Furthermore, Allen *et al.* [15] observed that the properties of the pure components such as viscosity and surface tension are higher for long-chain fatty acid esters than for medium-chain fatty acid esters. The physical properties compared in table 1 show higher viscosity and surface tension for methyl oleate. They also reported that the higher viscosity fuel shows poor atomization characteristics, larger droplet size and hence longer spray tip penetration. It can be concluded that the measured spray tip penetration of the biodiesel pure component follows in accordance with the difference in molecular characteristics and physical properties.

## 4. Numerical modelling of spray tip penetration

The spray models of the open-source OpenFOAM CFD code are used to simulate the pure component fatty acid ester spray. The modelling constants are tuned for the experimental condition of fuel injection pressure *P*_inj_=1000 bar and an ambient gas pressure *P*_amb_=30 bar. The KH-RT secondary break-up model and Rosin–Rammler distribution parameters are tuned for *n*-heptane which is a representative of diesel and methyl oleate and methyl laurate which are representatives of biodiesel [28,29]. One set of KH-RT model parameters is found to be valid for both methyl oleate and methyl laurate, whereas for *n*-heptane another set of parameters is used due to the difference in the break-up characteristics. The Rossin–Rammler distribution parameters are different for *n*-heptane, methyl oleate and methyl laurate as they represent three different categories of fuels.

**Figure 5.**
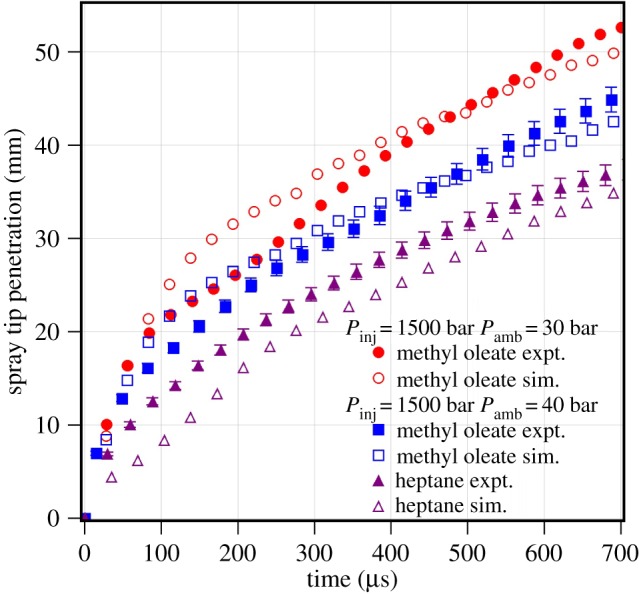
Comparison of measured and predicted spray tip penetration of methyl oleate and *n*-heptane at an injection pressure of 1500 bar.

**Figure 6.**
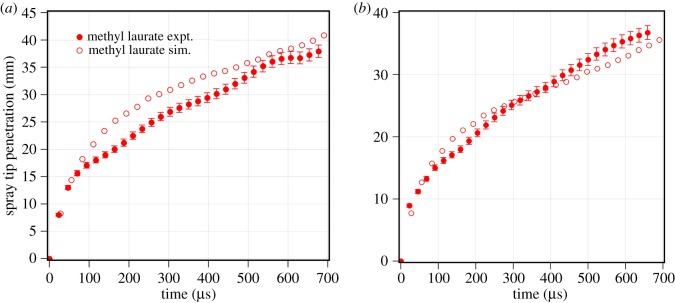
Comparison of measured and predicted spray tip penetration of methyl laurate. (*a*) *P*_inj_=1500 bar and *P*_amb_=30 bar. (*b*) *P*_inj_= 1500 bar and *P*_amb_=40 bar.

The objective is to validate the prediction of biodiesel single-component representatives with their corresponding measured results. The validation results for methyl oleate and *n*-heptane are shown in figure 5 at an injection pressure of 1500 bar and gas pressures of 40 bar and 30 bar. The predicted spray tip penetration of methyl oleate closely follows the measured spray tip penetration at both gas pressure conditions. The difference in the spray tip penetration during an initial part of spray injection is due to the initial transient nature of the spray and assumed fuel mass injection rate [30–32]. The predicted spray tip penetration for *n*-heptane closely matches with measurements. Similarly, the predicted spray tip penetration of methyl laurate is found to be in agreement with the measurements at both ambient gas pressures of 30 bar and 40 bar, as seen from figure 6. It can be concluded that the spray models are able to predict the spray tip penetration of pure component at different operating conditions within measurement error. These spray models are further used to predict the spray tip penetration of biodiesel and its blend with diesel.

### 4.1. Prediction of spray tip penetration of biodiesel

**Figure 7.**
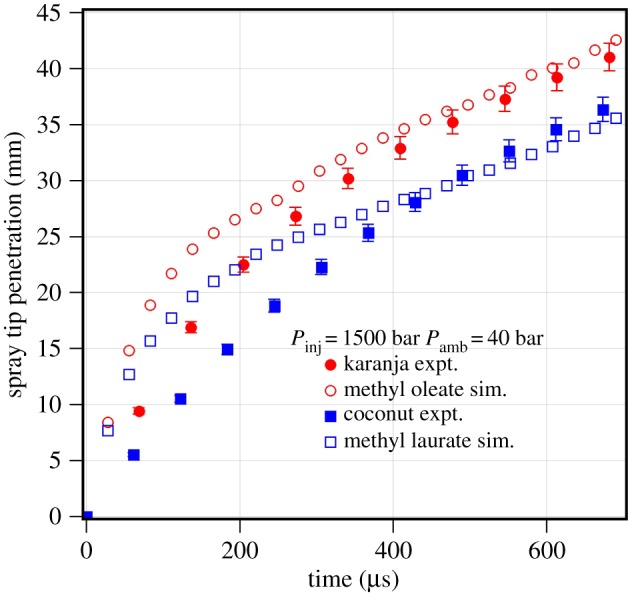
Experimental and predicted results for biodiesel at an injection pressure of 1500 bar and an ambient gas pressure of 40 bar.

Biodiesels, depending on their origin, have 8–10 different fatty acid esters [25]. The CFD simulation is performed with single representative component, whose physico-chemical properties are similar to those of biodiesel. In the literature, methyl oleate is commonly used as the representative fuel for various biodiesels irrespective of the composition of the biodiesel [27]. From the composition of various biodiesels, it is observed that karanja biodiesel has a high percentage of methyl oleate, and coconut and palm kernel biodiesels have a high percentage of methyl laurate [7]. The spray tip penetration of karanja and coconut is predicted with their single-component and multi-component representative fuels. The predicted results are shown for most common near top-dead-centre fuel injection event condition *P*_inj_=1500 bar, *P*_amb_=40 bar of the diesel engine. The comparison of measured and predicted results is shown in figure 7. It is observed that the predicted spray tip penetration of methyl oleate is similar to the spray tip penetration of karanja biodiesel. Similarly, the predicted spray tip penetration of methyl laurate has a close match with the measured spray tip penetration of the coconut biodiesel. Thus, it can be said that the spray tip penetration of commonly used biodiesels soya bean, rapeseed and karanja which have a high percentage of methyl oleate can be represented by methyl oleate. Similarly, non-evaporating spray of biodiesels of palm kernel and coconut can be represented by methyl laurate.

**Figure 8.**
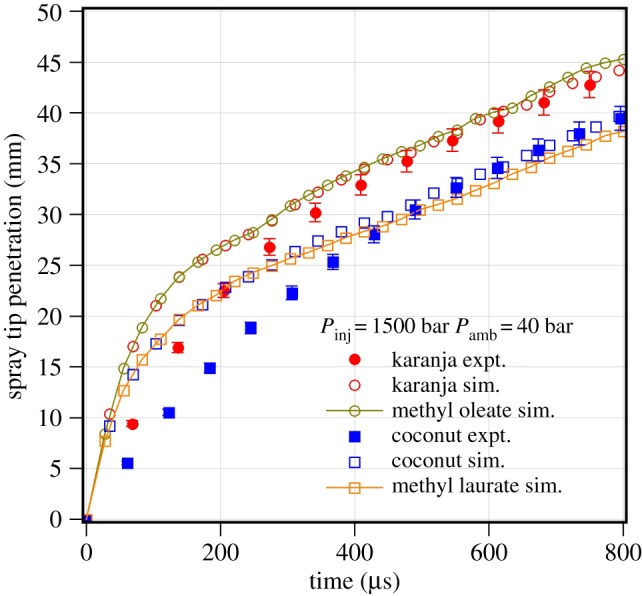
Experimental and predicted spray tip penetration of karanja and coconut biodiesel at an injection pressure of 1500 bar and an ambient gas pressure of 40 bar.

Figure 8 compares the measured spray tip penetration of biodiesel with predicted spray tip penetration using single-component and multi-component models. The karanja biodiesel is modelled with 5 FAME components in a proportion of methyl palmitate 5.94%, methyl stearate 5.44%, methyl oleate 61.34%, methyl linoleate 23.14%, methyl linolenate 4.14%. The coconut biodiesel is modelled with 5 FAME components in proportion of methyl laurate 53.82%, methyl myristate 21.22%, methyl palmitate 10.32%, methyl stearate 3.92% and methyl oleate 10.72%. These compositions are typical of respective biodiesel found commonly [24]. The spray model parameters are kept the same for both cases of single-component and multi-component representative simulations. The input to the multi-component model used in the study is the liquid mass fraction of five major components present in biodiesel. The biodiesel property is evaluated with mixture rule using physical properties of each pure component. The mixture rule for calculating physical properties of the biodiesel is given in equation (4.1):

4.1 $$\text{Property of biodiesel}=\sum_{n=1}^{5}(Y_{n}\times P_{n}),$$

where *Y*_*n*_=liquid mass fraction of pure components present in biodiesel, *n*=number of pure components present in biodiesel, *P*_*n*_=property of the *n*th component of biodiesel. It is observed that (figure 8) both the single-component and the multi-component representative gives similar spray tip penetration which further closely follows the measured spray tip penetration of their respective biodiesel. As the predicted spray tip penetrations of the single-component and multi-component representative are found to be similar for the biodiesel studied, it is concluded that the single component modelling for prediction of biodiesel spray tip penetration is sufficient instead of its multi-component representative under non-evaporating conditions.

### 4.2. Prediction of spray tip penetration of biodiesel blend with diesel

**Figure 9.**
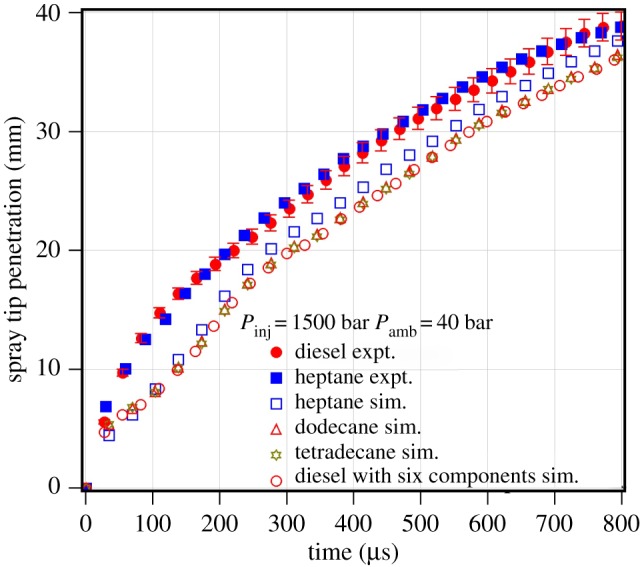
Experimental and predicted spray tip penetration of diesel, *n*-heptane, *n*-dodecane, *n*-tetradecane and six-component diesel representative at an injection pressure of 1500 bar and an ambient gas pressure of 40 bar.

Biodiesel is extensively used in blends with diesel. This avoids extensive engine modifications including injection system and fuel injection mapping. It is important to understand how the blending of biodiesel with diesel affects the spray characteristics. The spray tip penetration of 20% karanja biodiesel blend with diesel fuel is predicted with the single-component and multi-component representatives of diesel and biodiesel. The spray model is first validated with different single-component and multi-component representative of diesel. Figure 9 shows the validation of diesel fuel single component and multi-component representatives such as *n*-heptane, *n*-dodecane, *n*-tetradecane and six-component diesel representative. The multi-component representative of diesel contains six components such as toluene (15%), *n*-decane (14%), *n*-dodecane (22%), *n*-tetradecane (23%), *n*-hexadecane (13%) and *n*-octadecane (11%) [33]. Similar to biodiesel, the single-component and multi-component representatives of diesel are found to predict similar spray tip penetration. Thus, the use of single-component representative is also found to be sufficient for the prediction of non-evaporating spray tip penetration of diesel fuel. Among the three single component representatives studied, *n*-heptane, *n*-dodecane and *n*-tetradecane, *n*-heptane shows a spray tip penetration more close to that of the diesel fuel. Hence, *n*-heptane is selected as the single-component representative for diesel for blending with karanja biodiesel.

**Figure 10.**
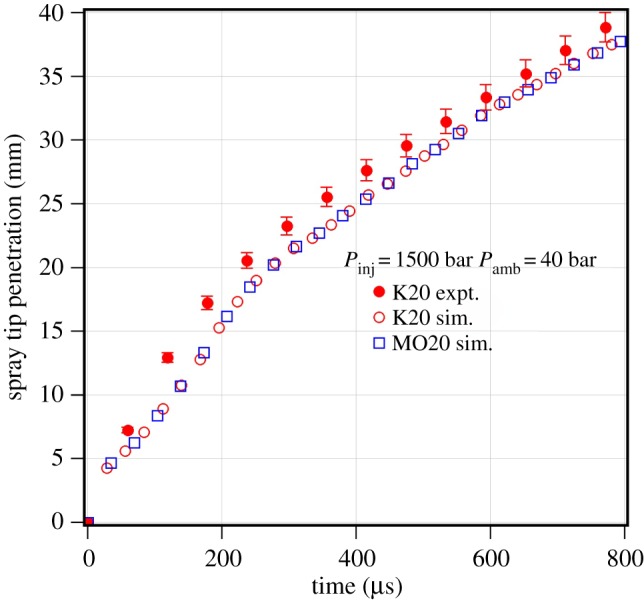
Experimental and predicted spray tip penetration of karanja biodiesel blend with diesel at an injection pressure of 1500 bar and an ambient gas pressure of 40 bar. (K20 = karanja biodiesel 20% blend with diesel; K20 sim = karanja biodiesel five-component representative 20% blend with *n*-heptane; MO20 = methyl oleate 20% blend with *n*-heptane.)

Figure 10 shows the comparison of predicted spray tip penetration of methyl oleate 20% blend with *n*-heptane and methyl oleate 20% blend with multi-component representative of karanja biodiesel, with the measured results of karanja biodiesel 20% blend with diesel fuel. It is observed that both the blends of the multi-component and single-component representatives of karanja biodiesel show similar spray tip penetration. This means that the blends can be predicted with the corresponding single-component representative for biodiesel.

## 5. Summary and conclusion

A comparative experimental and numerical study is conducted to establish the significance of single-component representatives for biodiesel, diesel and their blend for predicting their spray tip penetration. The representatives selected in the study categorize the biodiesels in two groups. Methyl oleate represents long-chain fatty acid ester biodiesels such as karanja, soya bean and rapeseed. Methyl laurate represents medium-chain fatty acid ester biodiesels such as coconut and palm kernel. The spray tip penetration of karanja biodiesel is predicted with corresponding single-component and multi-component representatives. The spray tip penetration of karanja biodiesel is found to be similar to that of methyl oleate. The observed similarity in the spray tip penetration of biodiesel is due to the presence of methyl oleate in its fatty acid ester composition. The spray tip penetration of coconut biodiesel is found to be similar to that of methyl laurate. Furthermore, the measured spray tip penetration of biodiesel and its 20% blend with diesel is also compared with the corresponding single-component and multi-component representatives which is found to be in good agreement with the measured spray tip penetration. It can be concluded that the single component representative of biodiesel is sufficient to predict the non-evaporating spray characteristics of biodiesel and its blend in the respective category. It is necessary to have the knowledge of the composition of the biodiesel and FAME component dominating physical properties of biodiesel to model the biodiesel spray.

## Data accessibility

Our data are deposited at Dryad: https://doi.org/10.5061/dryad.f7r7b [34].
